# Feeding Difficulties Following Vascular Ring Repair: A Contemporary Narrative Review

**DOI:** 10.7759/cureus.24623

**Published:** 2022-04-30

**Authors:** Danielle T Fisenne, Joseph Burns, Arushi Dhar

**Affiliations:** 1 Department of Pediatrics, Donald and Barbara Zucker School of Medicine at Hofstra/Northwell, Hempstead, USA; 2 Department of Pediatrics, Cohen Children's Medical Center, New Hyde Park, USA; 3 Department of Pediatric Cardiology, Cohen Children's Medical Center, New Hyde Park, USA

**Keywords:** congenital heart disease, pediatric cardiothoracic surgery, pediatric cardiology, feeding, vascular ring

## Abstract

Vascular rings are congenital abnormalities of the aortic arch vascular system that compress the trachea and esophagus. A review of long-term outcomes suggests that chronic feeding difficulties can persist following surgical repair of vascular rings. Previous reports of postoperative vascular ring division outcomes indicate that chronic esophageal symptoms may persist following repair, though most available data focuses on persistent respiratory symptoms. It is therefore the aim of this article to summarize and organize recent evidence reporting the frequency, presentation, and management of feeding difficulties following vascular ring repair in pediatric patients. Pathophysiologic mechanisms for postoperative esophageal symptoms may include residual compression from an unresected diverticulum of Kommerell or delayed repair leading to chronic esophageal dysmotility despite correction of esophageal compression. Guidance on the management of feeding difficulties following vascular ring repair is limited. The authors describe success in one case with nasogastric tube feeding and interdisciplinary evaluation. Consensus regarding the management of feeding difficulty following vascular ring repair is needed.

## Introduction and background

Vascular rings are congenital abnormalities of the aortic arch vascular system that can compress the trachea and esophagus. They originate as a result of aberrant embryological development of the aortic arch and its branches. A complete or true vascular ring contains both the trachea and esophagus, while an incomplete ring does not contain both structures [1]. 

Various malformations have been described and categorized. The most frequently reported are the double aortic arch, and the single right aortic arch with aberrant left subclavian artery and left-sided ductus arteriosus [1]. A diverticulum of Kommerell may be present in this latter type of ring, an outpouching from which the aberrant left subclavian artery may originate. Vascular rings comprise 1-2% of all congenital cardiac abnormalities [1]. Males are more often affected than females, with one study of 38 patients reporting a male to female ratio of 1.7:1 and another study of 82 patients reporting a male to female ratio of 2:1 [2,3].

Vascular rings in pediatric patients most commonly present with respiratory symptoms or feeding difficulty. In a sample of 64 patients with vascular rings, 91% had respiratory symptoms, which included inspiratory stridor, respiratory infections, cough, and respiratory distress [1]. 47% of patients had esophageal symptoms, the most common of which was dysphagia [1]. In another sample of 38 patients, symptoms indicative of esophageal compression were present in 50% of patients and included dysphagia, feeding difficulty, reflux, and vomiting. In 29% of patients, an associated cardiac anomaly was present [2]. Symptom presentation may vary according to the underlying abnormality. Patients with a double aortic arch typically present in the first year of life with more severe respiratory compromise due to significant tracheal compression. Patients with a single right aortic arch, aberrant left subclavian artery, and left ductus arteriosus instead present in the first few years of life with a combination of respiratory and esophageal symptoms [1]. 

Diagnostic evaluation of a vascular ring consists of several imaging modalities. For initial noninvasive evaluation, anterior and lateral chest radiographs are often performed [2]. Cardiovascular MRI/magnetic resonance angiography (MRA) and chest CT are the gold standard for diagnosis, are readily available, and can aid in pre-operative surgical planning by defining specific vascular anatomy [4]. An echocardiogram is also used in the preoperative evaluation of cardiovascular anatomy [4]. Direct laryngoscopy, bronchoscopy, and barium esophagram have been used to evaluate respiratory and esophageal symptoms, including recurrent respiratory infections and dysphagia, though these cannot elucidate vascular anomalies. Vascular rings diagnosed on a prenatal fetal echocardiogram are managed expectantly as it is difficult to determine the severity of tracheal or esophageal compression with this modality [5]. 

Treatment consists of surgical ring division repair. Current operative approaches include minimally invasive thoracoscopic repair and open thoracotomy. The choice of approach is dependent on the underlying anatomical malformation. In one sample of 82 patients undergoing vascular ring division, a left thoracotomy was used in 85% of cases. Right thoracotomy may be used in cases of an aberrant right subclavian artery. Repair of a double aortic arch is achieved by dividing the more hypoplastic of the two arches. In those with a right aortic arch with aberrant left subclavian artery and left ductus, the ligamentum or ductus is divided [3]. If a diverticulum of Kommerell is present, it may be resected with reimplantation of the aberrant left subclavian artery onto the left common carotid artery [6]. 

Outcomes of vascular ring division in pediatric patients have previously been described as excellent, but it is becoming increasingly evident that symptoms may persist following repair. As more studies have examined postoperative outcomes, chronic feeding difficulties and esophageal symptoms appear to persist in a number of patients despite initial improvement [2]. Though several studies have reported on persistent respiratory symptoms following repair, few have focused on persistent feeding difficulties. In addition to the lack of reported data regarding the frequency of this issue, guidelines for the management of affected patients are scarce. Organized data and evidence-based guidance are needed for providers who manage patients with chronic feeding difficulty after vascular ring repair. Although the authors include data on postoperative respiratory symptoms, it is the aim and focus of this article to summarize and organize recent evidence reporting the frequency, presentation, and management of feeding difficulties following vascular ring repair in pediatric patients.

## Review

A comprehensive literature review was conducted utilizing the PubMed and PubMed Central databases. A range of keywords was used to identify relevant studies regarding pediatric vascular ring repair outcomes, including “pediatric vascular ring,” “vascular ring repair,” and “vascular ring outcomes.” Selection criteria for studies cited in this review included studies published in peer-reviewed journals that were original articles, review articles, or case reports. A total of 19 peer-reviewed articles were selected for inclusion in this literature review, with data included from 2001 to 2021.

Feeding difficulties following vascular ring repair

It is estimated that as many as 45% to 65% of patients may have persistent feeding or respiratory symptoms following vascular ring repair [7]. Among patients with recurrent symptoms, common feeding challenges that have been reported include dysphagia with solid foods or cough [6]. Postoperative dysphagia and cough are best evaluated by CT with three-dimensional reconstruction and barium esophagram [6]. 

Persistent feeding difficulty following repair has been reported across a range of time postoperatively, from hospital discharge to 6.8 years after repair [8,9]. A Danish study of 23 patients with a median follow-up of 6.8 years reports that early post-operative malnutrition was common. Normal eating habits were present in just 50% of patients at three months after repair [8]. After more than one year, 14% of cases were still underweight. In long-term follow-up, though feeding is not explicitly reported, only 14% of patients were asymptomatic. However, the authors do report that the majority of complaints were respiratory [8]. 

In a different sample of 62 patients, of whom 20 had a dysphagia component prior to repair, all had resolution of swallowing difficulties at the time of discharge. Among all patients during the follow-up period, no residual symptoms were reported in 63% of patients at one month or in 82% of patients at six months after repair. However, this study does not discern between respiratory and feeding symptoms [9]. 

Fewer studies have reported specifically on feeding outcomes independent of respiratory symptoms after repair. These studies also report a range of follow-up time. In one study of 63 patients, 40 of whom had a single aortic arch and 23 had a double aortic arch, feeding difficulty was reported in 62.5% of single arch cases and 48% of double arch cases. At a median follow-up of 17.4 months, 17.5% of single arch patients and 13% of double arch patients had persistent swallowing difficulties. Further, at 36 months, five out of 10 single arch respondents in a phone survey continued to report swallowing challenges. This team contends that vascular ring division may only partially relieve associated feeding difficulties [10]. Although there was no significant difference in age at the time of repair or in rates of persistent feeding difficulty between patients with single or double arch anatomy, further study is needed to elucidate whether the type of vascular anomaly is associated with the persistence of symptoms.

In an evaluation of 38 patients, in which 19 had dysphagia, chronic issues related to feeding persisted in 16% of patients at four to six weeks postoperatively. Of these patients, one had a tracheoesophageal fistula, and two had genetic syndromes with associated gastroesophageal reflux. Repair at greater than three years of age was associated with persistent symptoms in fewer patients (50%) than in those who underwent repair prior to six months of age (80%). It was noted, however, that chronic symptoms in the early repair group were respiratory. This team suggests that the need for early repair may reflect more severe underlying pathology that contributes to greater persistence of symptoms postoperatively [2].

Not all studies describe a frequency of symptom recurrence as high as those previously discussed. Though it does not specify whether the recurrence was of primary respiratory or feeding symptoms, a recent article describes only two cases of persistence or recurrence among 58 vascular ring repairs [11]. An evaluation of 148 patients, 35% of whom had dysphagia at presentation, reports recurrent or persistent symptoms in only 14 cases at a median follow-up of 1.72 years. Though the exact number with gastrointestinal symptoms is not reported, these patients are described as endorsing “occasional dysphagia” [12].

One study reports improvement of postoperative feeding difficulties following a second operative intervention. This interesting report describes patients with a right aortic arch and Kommerell’s diverticulum with persistent symptoms requiring re-operation. After initial repair, four out of 12 patients had recurrent dysphagia requiring resection of the diverticulum and left subclavian artery transfer. Each of these patients had resolution of symptoms with re-operation [13]. 

Several studies comment on a possible pathophysiologic basis for persistent feeding difficulties despite surgical correction. One team promotes resection of the diverticulum of Kommerell in vascular ring repair when possible, as this structure may lead to residual esophageal compression [6]. Alternatively, delayed surgical correction may result in chronic esophageal dysmotility due to abnormal innervation and growth of the esophagus secondary to compression during development [2]. It is likely that feeding difficulties in the setting of vascular rings are not exclusively due to compression, as is suggested by a report of one case in which an underlying neurocristopathy may have contributed to both vascular ring formation and long-term symptoms following repair. This furthers the contention regarding the necessity of repair in children with a vascular ring and chronic feeding difficulty, as surgical repair may not definitively resolve symptoms [14].

Previous studies that have investigated postoperative feeding protocols have examined patients with single or biventricular congenital heart defects rather than those with vascular rings [15,16]. These studies also focus solely on implementing feeding protocols in the immediate postoperative period in critical care units rather than on feeding difficulties that persist long after repair [17,18]. These studies do agree that optimizing perioperative nutrition in patients with congenital heart disease is important for achieving appropriate growth and development [19].

The lack of consensus on the management of feeding difficulty after vascular ring repair presents challenges for the pediatricians who care for patients with this issue. Current practices vary among institutions, and available guidance is limited. The authors had success in a single case of a four-month-old female with postoperative ring repair feeding intolerance using interdisciplinary evaluation from speech, dietary, and gastrointestinal teams. The patient’s previously repaired ring abnormality was a single right aortic arch with left-sided ductus arteriosus and aberrant left subclavian artery originating from a prominent diverticulum of Kommerell. She presented with cough and vomiting after bottle-feeding at one week postoperatively from a left thoracotomy with the division of the left ductus and reimplantation of the aberrant left subclavian artery onto the left carotid artery. Diagnostic evaluation postoperatively consisted of modified barium swallow, which revealed esophageal dysmotility, laryngeal penetration, and trace aspiration. Feeding intolerance was managed with nasogastric tube bolus feedings of expressed human milk fortified with NeuroPro formula (Enfamil, Chicago, Illinois) at 22 kcal/oz to maintain a nutritional intake of at least 100 kcal/kg/day. Volume and rate of feeds were adjusted according to the patient’s ability to tolerate this regimen without dysphagia, coughing, or vomiting. The patient was discharged home on the above nasogastric tube feeding regimen with a plan to continue feeding management and weight monitoring under the guidance of her general pediatrician. Additional outpatient follow-up included swallow therapy. 

## Conclusions

It is evident from recent literature that feeding difficulties can persist following vascular ring repair. This review reflects a broad range of reported rates of persistence. Given these varying frequencies of symptom persistence, clear associations to variables such as follow-up time, type of vascular anomaly, and method of surgical approach are limited. Duration of follow-up after repair particularly spans across a broad range of time among the studies examined in this review. This may contribute to the varying reported rates of persistence of symptoms. The heterogeneity in the type of vascular rings reported may also render certain studies non-generalizable to the general population. 

Additional study is needed to further elucidate possible associations between chronic feeding difficulty and age at the time of repair. As with follow-up time and frequency of symptom persistence, the median age at the time of repair was also reported across a broad range. Given that several studies included patients with associated anatomical anomalies or genetic syndromes, it would also be interesting to examine the relationship between persistent symptoms and the presence or absence of such anomalies. 

Further guidelines are needed regarding the evaluation and management of feeding difficulties that persist in pediatric patients following vascular ring repair, as most studies on postoperative outcomes report solely on respiratory symptoms. Though the authors had success managing this issue with interdisciplinary follow-up and nasogastric tube feeds, little data is available to assist pediatricians in choosing optimal feeding strategies for affected patients. As feeding difficulties following ring repair are further investigated, it is the authors’ aim that the data summarized in this review will encourage the necessary development of standardized feeding management guidelines for this issue. 
